# Temporal and spatial lags between wind, coastal upwelling, and blue whale occurrence

**DOI:** 10.1038/s41598-021-86403-y

**Published:** 2021-03-25

**Authors:** Dawn R. Barlow, Holger Klinck, Dimitri Ponirakis, Christina Garvey, Leigh G. Torres

**Affiliations:** 1 Geospatial Ecology of Marine Megafauna Lab, Marine Mammal Institute, and Department of Fisheries and Wildlife, Oregon State University, Newport, OR USA; 2 Center for Conservation Bioacoustics, Cornell University, Ithaca, NY USA; 3 Marine Mammal Institute, Department of Fisheries and Wildlife, Oregon State University, Newport, OR USA; 4 University of Maryland, College Park, MD USA

**Keywords:** Ecology, Ocean sciences, Marine biology, Physical oceanography, Ecology

## Abstract

Understanding relationships between physical drivers and biological response is central to advancing ecological knowledge. Wind is the physical forcing mechanism in coastal upwelling systems, however lags between wind input and biological responses are seldom quantified for marine predators. Lags were examined between wind at an upwelling source, decreased temperatures along the upwelling plume’s trajectory, and blue whale occurrence in New Zealand’s South Taranaki Bight region (STB). Wind speed and sea surface temperature (SST) were extracted for austral spring–summer months between 2009 and 2019. A hydrophone recorded blue whale vocalizations October 2016-March 2017. Timeseries cross-correlation analyses were conducted between wind speed, SST at different locations along the upwelling plume, and blue whale downswept vocalizations (D calls). Results document increasing lag times (0–2 weeks) between wind speed and SST consistent with the spatial progression of upwelling, culminating with increased D call density at the distal end of the plume three weeks after increased wind speeds at the upwelling source. Lag between wind events and blue whale aggregations (n = 34 aggregations 2013–2019) was 2.09 ± 0.43 weeks. Variation in lag was significantly related to the amount of wind over the preceding 30 days, which likely influences stratification. This study enhances knowledge of physical-biological coupling in upwelling ecosystems and enables improved forecasting of species distribution patterns for dynamic management.

## Introduction

A central pursuit of ecology is to decipher and understand the complex relationships between biological organisms and their physical environment. Coastal upwelling zones are coupled physical-biological systems that lead to some of the greatest productivity on earth, and account for over 20% of marine fisheries catches despite covering an area less than 5% of the global oceans^1–3^. The upwelling process is fueled by winds that drive a net movement of surface water offshore^4^, which is replaced by cold, nutrient-rich water that supports elevated primary productivity once transported into the photic zone. This primary productivity sustains high densities of forage species such as zooplankton and fish, which lead to feeding opportunities for marine top predators. Although many of these physical and biological connections in upwelling systems are documented, mismatches exist due to spatial and temporal lags between physical drivers, primary production, prey availability, and predator distribution patterns^5^.

Many marine predator populations occupy upwelling systems^6^ where they are threatened by human activities in that region^7^, emphasizing the need for greater understanding of their ecology to inform effective management. The distribution patterns of these highly mobile species reflect dynamic ocean processes. Therefore, management efforts to effectively protect marine predator populations should also be dynamic in space and time^8–12^. Physical characteristics of the marine environment, such as temperature, are frequently used to describe upwelling conditions and predict marine predator distribution *e.g.*^7,13–15^. The responses of marine predators to upwelling conditions are predominantly indirect^16^, facilitated through correlations with increased prey availability that results from enhanced upwelling and primary productivity. However, the inherent lag between physical drivers of upwelling (*e.g.*, wind patterns) and biological responses are rarely explicitly tested or incorporated into species distribution models. Therefore, to maximize the predictive capacity of species distribution models and consequently increase the efficacy of dynamic management efforts, information on the spatial and temporal lags along this coupled physical-biological pathway is needed.

Throughout the world blue whales (*Balaenoptera musculus*) rely on coastal upwelling systems for foraging, where elevated primary production and prey density supports energetic needs^17–21^. As the largest animal on earth, blue whales have intense energetic demands^22,23^, and are selective predators of krill^24–26^. On seasonal and inter-annual scales, blue whale migratory movements are known to lag annual spring blooms in primary production^27,28^, and track the distribution patterns of their krill prey^24,29,30^. While this coupled physical-biological pathway from wind to whales^24^ has been explored on broad scales and seasonal cycles, the within-season lags along this mechanistic pathway have seldom been robustly explored or quantified at a fine temporal resolution.

The relationship between wind speed and biological production in upwelling systems is variable around the world. In eastern boundary current Ekman-type upwelling systems, such as those found off the west coast of the United States, Peru, and West Africa, a dome-shaped relationship exists between wind speed and productivity. In these eastern boundary current systems productivity increases until a certain wind speed is reached, after which point it decreases with increasing wind speed due to advection of nutrients or turbulent mixing^31–33^. This “optimal environmental window” for maximum biological productivity occurs at wind speeds of 10–12 m s^−1^ for primary productivity, and 5–6 m s^−1^ for fish recruitment^31,33^. Temporal variability in upwelling winds (*e.g.* intermittent periods of relaxation between upwelling events) further contributes to maximizing biological production^34^ in these Ekman-type upwelling systems. Conversely, in non-Ekman-type and low-wind upwelling systems, the relationship between upwelling intensity and biological productivity appears to be linear^33,35^. In the Bonney upwelling off the south coast of Australia, mean wind speeds are only 3 m s^−1^, and increasing wind speeds lead to enhanced phytoplankton productivity^35^.

**Figure 1:**
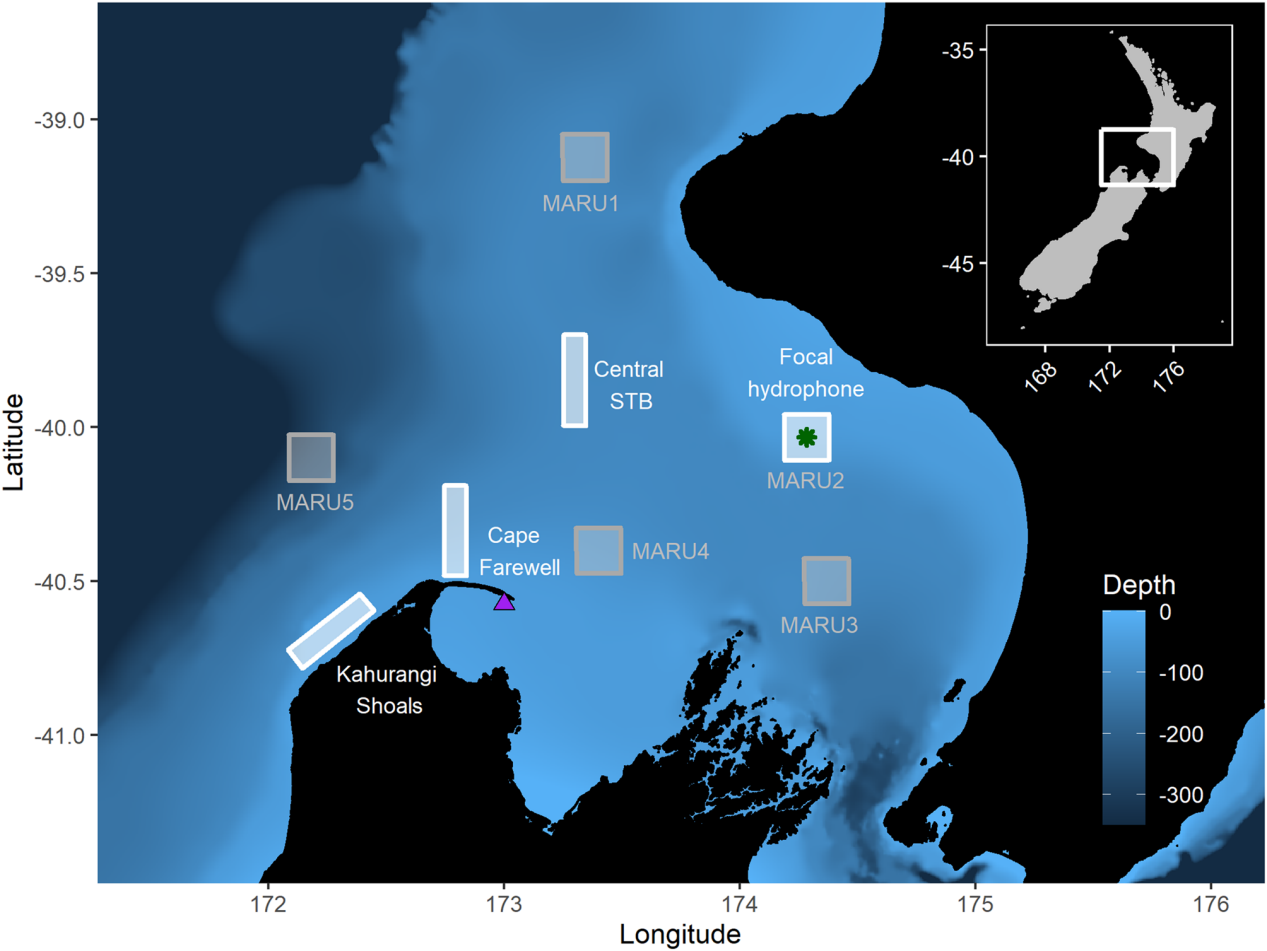
Map of the study region within the South Taranaki Bight (STB) of New Zealand, with location denoted by the white rectangle on inset map in the upper right panel. All spatial sampling locations for sea surface temperature are denoted by the boxes, with the four focal boxes shown in white that represent the typical path of the upwelling plume originating off Kahurangi shoals and moving north and east into the STB. The purple triangle represents the Farewell Spit weather station where wind measurements were acquired. The location of the focal hydrophone (MARU2) is shown by the green star.

Coastal upwelling is evident in the South Taranaki Bight region of New Zealand^36^ (STB; Fig. 1), which lies between the country’s North and South Islands. Off the northwest coast of the South Island, the intermittent Westland Current is intensified by westerly winds, creating a sea surface slope that accelerates deep water movement over the shallow bathymetric feature of Kahurangi Shoals. The result is strong upwelling off Kahurangi point, and the formation of a plume of cold, saline, nutrient-rich water that moves northeast, around Cape Farewell and eastward through the STB, carried by the D’Urville Current^37,38^. The upwelling plume supports enhanced primary production relative to surrounding waters, particularly during spring and summer months when the water column is more stratified and surrounding waters are presumably nutrient-depleted^39^. While upwelling events occur at all times of the year, the cold surface signature of the plume is most visible during the spring and summer months (October–March) when it can be clearly detected via satellite imagery^39^ (Supplementary Information, Fig. S1). Although the Kahurangi upwelling is an Ekman-type upwelling system, productivity is not constrained by shelf width or advection offshore. The upwelling plume supports the presence of zooplankton aggregations, including the krill species *Nyctiphanes australis*^40,41^. The STB region is a foraging ground for a population of pygmy blue whales (*B. m. brevicauda*) that is genetically distinct and present in the region year-round^42,43^. Physical properties of the water column structure in the Kahurangi upwelling system, including cooler surface temperatures and sub-surface water column features (*e.g.* mixed layer depth, thermocline temperature, thermocline strength), lead to enhanced krill availability, and consequently increased foraging opportunities for blue whales in the summertime^21,44^.

In this study, data on wind, remotely sensed sea surface temperature (SST), and blue whale occurrence patterns derived from visual and acoustic observations are compiled to evaluate physical-biological coupling in an upwelling system and quantify the lags between wind forcing and biological response by an upper trophic level predator. Timeseries analyses are used to test the hypothesis of lags between wind input, decreased temperatures at different spatial locations along the path of the upwelling plume, and elevated blue whale occurrence patterns. We anticipate that quantifying lag time between wind input and marine predator distribution patterns will improve spatial dynamic management efforts in the STB by extending forecasting capacity via longer lead times and refined spatial predictions. While our methods are developed and implemented for the STB ecosystem, they are transferable to other upwelling systems to inform, assess, and improve predictions of marine predator distributions by incorporating lag into our understanding of dynamic marine ecosystems.

## Methods

### Remotely sensed data sources

Daily wind speed and direction were measured at a shore-based weather station located on Farewell Spit in the STB (Fig. 1). All wind data were downloaded from New Zealand’s National Climate Database (https://cliflo.niwa.co.nz/), and accessed via the ‘clifro’ package in R^45^. Wind speed and direction measurements were recorded daily at 0900 local time, and these measurements were downloaded for a 10-year timeseries between 1 January 2009 and 31 December 2019. A circular-linear correlation^46^ was run to test the relationship between wind speed and direction during the austral spring and summer months (October–March).

Daily SST data were extracted at eight designated spatial sampling locations (“boxes”) throughout the study area (Fig. 1). Four of these boxes were selected for the presented analysis and results as they capture the typical path of the upwelling plume^39^ (Fig. 1, white boxes) from the upwelling source over Kahurangi Shoals to downstream locations within the STB of Cape Farewell, Central STB, and the location of a focal hydrophone (“MARU2”; see “Methods”: “Acoustic data” for details). Each box has an area of 256 km^2^. Multi-scale Ultra-high Resolution (MUR) SST data were downloaded at a 0.01 degree spatial resolution and daily temporal scale from the ERDDAP server using the rerddapXtracto package in R^47^. SST data were extracted for the same 10-year timeseries as the wind data at all eight box locations.

### Acoustic data

Acoustic data were recorded using a Marine Autonomous Recording Unit (MARU)^48^ deployed at 66 m depth in the STB (Fig. 1) as part of a broader effort to understand spatiotemporal occurrence patterns of blue whale vocalizations in the region^43^. The hydrophone had a flat frequency response (± 2.0 dB) in the 15 to 585 Hz band and recorded continuous acoustic data at a 2 kHz sampling rate with a high-pass filter at 10 Hz and a low-pass filter at 800 Hz. Acoustic data recorded between 1 October 2016 and 31 March 2017 were used for this study to document whale presence in the area, with a brief gap in recording between 28 December 2016 and 6 January 2017 for hydrophone refurbishment.

Downswept vocalizations known as D calls (~ 100–20 Hz) are produced by blue whales of both sexes on feeding grounds, and are considered social or contact calls that potentially serve to attract other blue whales to feeding areas or maintain group cohesion during feeding^49^. Spectral characteristics of blue whale D calls have been described for recordings obtained around the world, including in New Zealand waters^50^. Putative D calls were identified in the recordings using a spectrogram template correlation detector^51^, implemented in the acoustic analysis program Raven Pro 2.0^52^. Template spectrograms of 13 example D calls were selected to capture the variability in the call type. All template spectrograms had a 2 kHz sampling rate and were generated using a 2048 sample Hann window (3 dB filter bandwidth: 1.40 Hz) with 50% overlap. The detection threshold was empirically evaluated and set to 0.8, and each putative D call identified by the detector was compared against the template with the highest spectrogram correlation score. Detector performance was evaluated on a subset of 12 recording days that were manually reviewed and annotated for D call occurrence; manual annotations were considered true detections. Detections were considered true positives if they overlapped with a manually annotated call by at least 50% in time and frequency, and precision (proportion of detections that were true detections), recall (proportion of true calls that were detected correctly) and the number of false positives per hour were calculated using custom MATLAB scripts^53,54^. After running the detector on the full recording period of interest, all detection events were manually reviewed by an analyst (DRB) in Raven Pro 1.6, and false positives were removed prior to further analysis.

Sound propagation modeling was conducted to standardize the number of D call detections by the listening range of the hydrophone unit (D call density). The Range-dependent Acoustic Model (RAM)^55^ was used to estimate the hourly detection range at the hydrophone by simulating call propagation from source to receiver under the conditions at the recording time. RAM incorporates the sound speed profile within the water column, source depth, receiver depth, and seafloor substrate characteristics, all of which influence the propagation of sound and therefore impact the detection range for calling blue whales. Sound speed profiles were based on the World Ocean Atlas (WOA2009), bathymetry was extracted at a 1-arc minute resolution from the ETOPO1 dataset^56^, and geo-acoustic parameters for fine sand^57,58^ (grain size phi = 3) were used in the propagation model. The source depth (depth of the calling whale) was set to 5 m below the surface, the source level of the calls was set to 174 dB^59^. D calls were simulated at varying distances along 8 radials centered on the hydrophone, and detection range was estimated given the ambient noise levels recorded at the hydrophone. Sources of ambient noise that could contribute to variable detection range include tidal current flow noise, weather events, vessel traffic, and seismic airgun operation. For the ambient noise levels, the 95th percentiles of the 20–100 Hz band level (dB re 1 µPa^2^) were computed, as these values represent the sound level exceeded 5% of the time and therefore captures chronic, background noise levels as well as some episodic noise sources, such as seismic airgun activity. Detection area was calculated using the estimated detection distance along each bearing from the receiver. The propagation model was run at an hourly scale and summarized to obtain a daily mean detection area. Finally, a standardized estimate of daily D call density was obtained by dividing the number of D call detections per day by the daily mean detection area.

### Timeseries analysis

The 10-year timeseries of daily wind speed and SST at all locations were first examined for autocorrelation. The partial autocorrelation function was applied, which evaluates temporal autocorrelation within a timeseries by measuring the correlation between a timeseries and a time-shifted version of itself. To reduce detected autocorrelation due to the daily scale of the timeseries data, all datasets were subsampled to weekly increments. For wind speed, a weekly mean value was applied. For SST and D call density, each timeseries was subsampled to include only one daily measurement per week spaced at even increments (*i.e.*, daily measurements were taken every 7th day).

The focus of this study is on pulsed wind-driven upwelling events, and therefore the timeseries were first decomposed to remove long-term trends and seasonal effects, enabling the isolation of the stationary process of interest^60^. Furthermore, the emphasis of this study is on upwelling dynamics in the austral spring and summer months when the upwelling signal is strongest^39^, so the timeseries data were subset to only include the period between 1 October and 31 March of each year. All subsequent analyses were conducted using the detrended timeseries for the austral spring and summer months of each year between 2010 and 2018 (2009 and 2019 were dropped in the decomposition and calculation of the trend). Due to the shorter time span of the D call density timeseries (less than one full year), these data could not be decomposed; therefore, the observed values were used without decomposition for further analysis.

Correlation and lag were quantified between all timeseries data representing different spatial and temporal steps along the path of the upwelling plume (all eight boxes). The cross-correlation function (CCF) examines the correlation between two timeseries at different time lags^60^. For example, correlation between SST and wind speed in real time, between SST and wind speed one week prior, between SST and wind speed two weeks prior, etc*.* The CCF analysis produces a measure of correlation (autocorrelation function value, ACF) for each time step (lag, measured in weeks) that ranges between − 1 and 1, where values < 0 represent negative correlations and values > 0 represent positive correlations. The lag at which the timeseries are most strongly correlated represents the dominant time lag between the underlying physical and biological processes in the system. CCF analysis was conducted separately for each spring–summer period (eight years), and subsequently the mean and variance were calculated across all years analyzed for each relationship. Two documented regional marine heatwaves occurred during the study period in 2016 and 2018^61^; hence, these years were not included in the calculation of the mean lag and correlation values, and were instead examined separately. While this analysis does not explicitly test spatial lags *e.g.*^62^, temporal lag analysis was conducted between strategically chosen spatial locations to capture lag along the spatial progression of the upwelling plume.

### Timing of blue whale aggregations

To extend and quantify the relationship between pulsed wind events and blue whale foraging opportunities, the lag time between wind events and aggregations in blue whale sightings in the STB was estimated. Blue whale sighting reports were compiled between 2013 and 2019 from the marine mammal sightings database curated by the New Zealand Department of Conservation, sightings reported by marine mammal observers aboard seismic survey vessels, and from a few dedicated research efforts in the region^43,63,64^. These sightings data are treated as presence-only data because most sighting reports were opportunistic. Therefore, aggregations of sightings were identified to increase confidence in the biological relevance of blue whale sighting locations and minimize bias from the opportunistic nature of the dataset. Aggregations were defined as five or more whales seen within one week, and within 50 km, of one another.

To examine the relationship between wind and blue whale sighting aggregations, “wind events” were defined as two or more consecutive days where daily wind speed exceeded the mean spring–summer wind speed in the region (5.5 m s^−1^). Mean wind speed in the austral spring and summer (October–March) was calculated from wind speeds measured at the Farewell Spit weather station between 2009 and 2019. The Kahurangi upwelling is not an eastern boundary current system, and increased wind speeds will not lead to advection of nutrients beyond a continental shelf; rather, stronger winds lead to the formation of a stronger, colder upwelling plume in the STB^39^. Therefore, despite the characterization of the Kahurangi upwelling as an Ekman-type system, we assume that the relationship between wind speed and productivity is linear, similar to the Bonney upwelling off southern Australia^35^.

The lag between wind events and blue whale aggregations was calculated as the difference in days between the aggregation start date and the start date of the most recent previous wind event. The mean and standard error of the lag were computed across all sighting aggregations. Detected variability in lag time between wind events and whale aggregations was hypothesized to be a function of how well-mixed the ecosystem already is prior to the proximate wind event; *i.e.*, lag times are shorter when the system is already well-mixed by previous, recent wind events. To test this hypothesis, a generalized linear model (GLM) was fit between the lag time in days and the number of days in the preceding 30 days where wind speed was above the wind event threshold. The model was fit with a Poisson distribution, suitable for count data, using the ‘stats’ package in R. To examine the impact of the wind speed threshold used to define wind events, a sensitivity analysis of GLM results was conducted comparing the lag between blue whale aggregations and wind events defined by four different wind speed thresholds. The thresholds tested spanned the range of measured wind speed values during the study period and included the mean value (3.0, 5.5, 8.0, and 10.5 m s^−1^).

## Results

### Remotely sensed data

The circular-linear correlation analysis of wind speed and direction revealed a highly significant relationship, whereby stronger wind speeds are more likely to be from a westerly direction (p = 3.27*10^–80^; Supplementary Information, Fig. S2). Therefore, all further analyses were carried out with the assumption that in the spring and summer, higher wind speeds originate from westward of the Farewell Spit weather station. The timeseries decomposition revealed a highly seasonal cycle in SST and wind speed, along with a 10-year trend of increasing SST at all locations (Supplementary Information, Fig. S3).

### Acoustics

**Figure 2:**
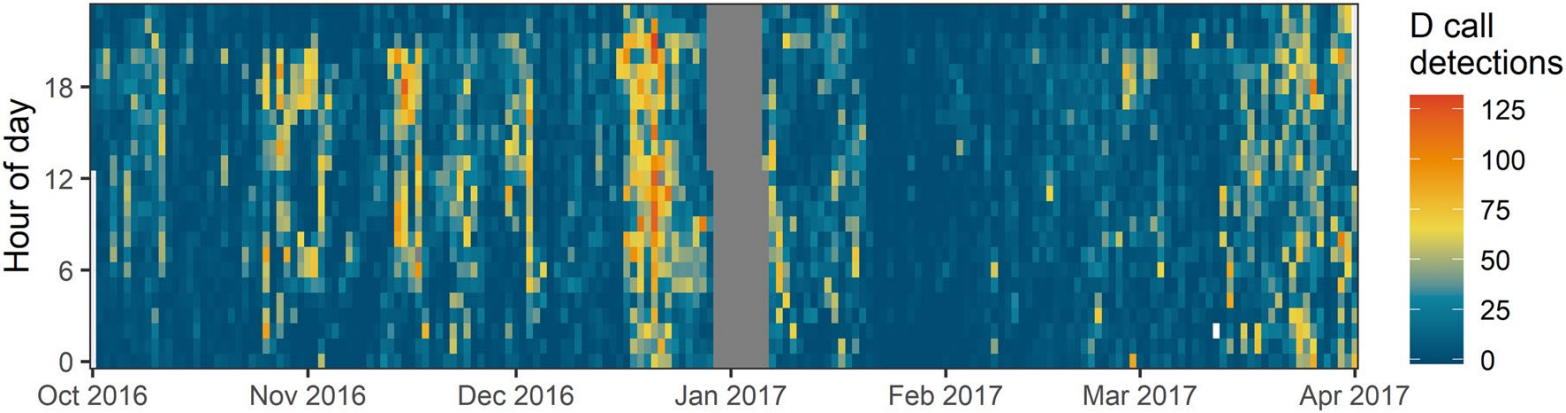
Heatmap showing the number of D call detections per hour at the MARU2 hydrophone between 1 October 2016 and 31 March 2017. Note pulsed increases in D call detections.

The D call detector evaluation produced a precision score of 0.51, recall of 0.81, and a false alarm rate of 13.22 false positives per hour. Once false positives were manually removed, pulses in D call detections were visually evident over the recording period (Fig. 2). The daily detection range of the hydrophone was relatively small (mean distance = 5.5 km, SD = 4.2, maximum distance = 34.8 km; measurements averaged across all 8 radials). The estimated daily detection area of the hydrophone ranged, depending on varying local ocean noise conditions, between 2.30 km^2^ and 5060.49 km^2^ over the recording period (mean = 219.58 km^2^). These detection results indicate that the majority of D calls recorded by the hydrophone were produced by blue whales close to the recording location.

### Timeseries cross-correlation analysis

**Figure 3:**
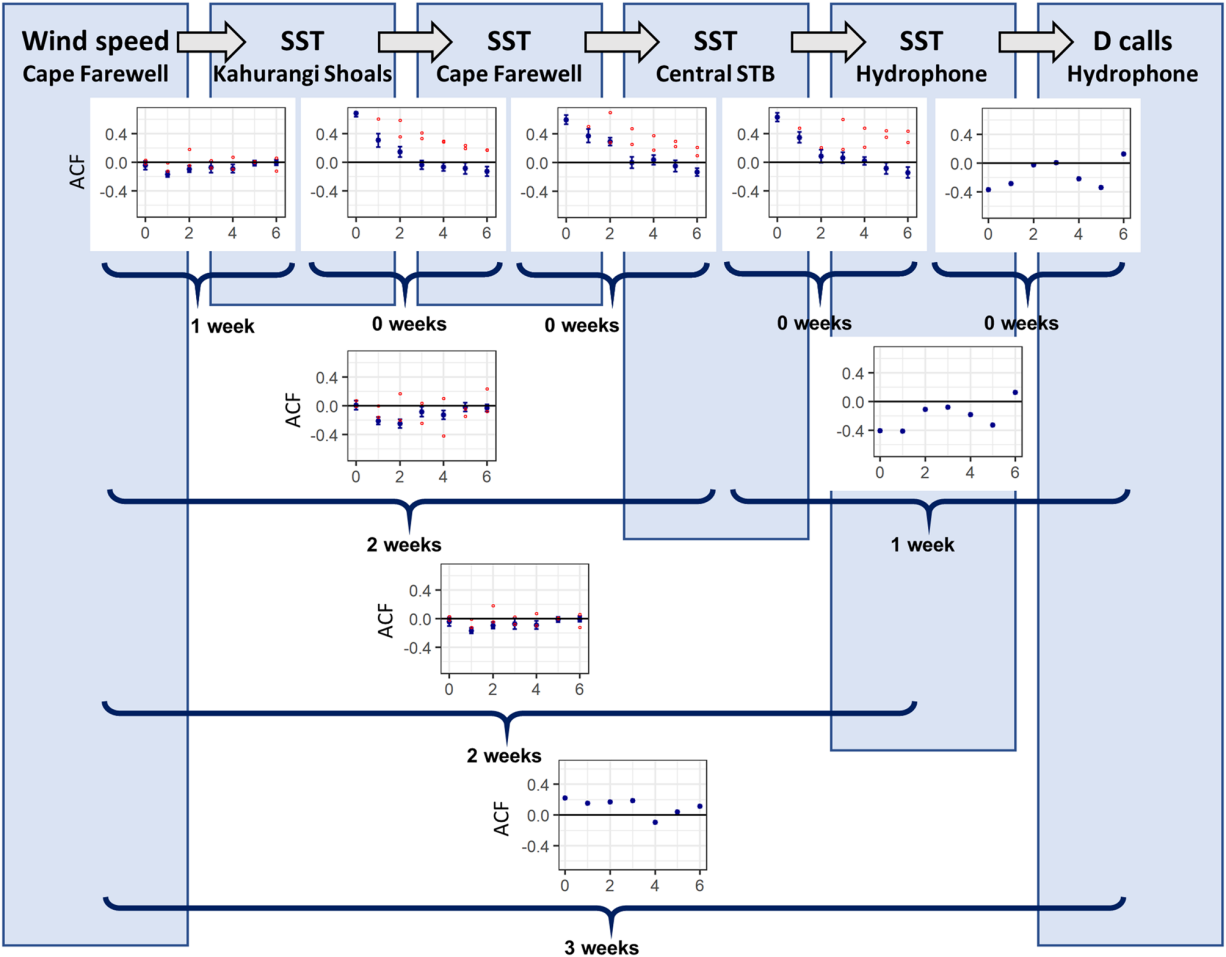
Results from the cross-correlation analysis along the typical path of the upwelling plume and schematic of time lags. Lag values are reported in weeks (x-axes). The autocorrelation function (ACF) measures the strength of the correlation between timeseries at that lag value, with values > 0 representing positive correlation, and values < 0 representing negative correlation (y-axes). Mean ACF was calculated at each lag step for all years between 2010 and 2018, with 2016 and 2018 partitioned a priori because of documented marine heatwave conditions. The ACF values are plotted in blue for “typical” conditions (mean ± standard error), and values from known heatwave years are plotted in red. D call density was only calculated in 2017, therefore a single ACF value is reported for each lag step.

The timeseries cross-correlation analysis revealed that wind speed, SST at different boxes along the path of the upwelling plume, and blue whale D call density were most correlated at different lag times depending on their spatial proximity (Fig. 3, Table 1; Supplementary Information, Table S1). [A supplementary analysis to assess the impact of the selected start day of SST weekly subsampling revealed no impact on the cross-correlation results; Supplementary Information, Fig. S4]. SST was positively correlated between all boxes at a lag of less than one week, and these relationships showed the strongest correlations (Table 1). Wind speed had the strongest negative correlation with SST at Kahurangi at a 1-week lag, and at a 2-week lag for the boxes further downstream along the path of the upwelling plume. D call density showed a negative correlation with SST at all boxes (*i.e.*, lower temperatures correlate to higher D call densities), and the strongest correlation occurred at a 3-week lag from SST at Kahurangi, at a 1-week lag from SST at Cape Farewell and Central STB, and at less than a week relative to SST at the hydrophone location. Finally, there was a positive correlation between wind speed and D call density at a 3-week lag (*i.e.*, higher wind speeds correlate to higher D call densities three weeks later).

**Table 1:** Results from the cross-correlation analysis between timeseries data from the four focal boxes.

	SST_Kahurangi_		SST_Cape Farewell_		SST_Central STB_		SST_hydrophone_		Dcalls_hydrophone_	
	Lag	ACF	Lag	ACF	Lag	ACF	Lag	ACF	Lag	ACF
Wind speed	1	− 0.17 ± 0.04	2	− 0.23 ± 0.05	2	− 0.25 ± 0.06	2	− 0.21 ± 0.07	3	0.19
SST_Kahurangi_			0	0.69 ± 0.04	0	0.57 ± 0.05	0	0.49 ± 0.08	3	− 0.32
SST_Cape Farewell_					0	0.60 ± 0.07	0	0.53 ± 0.07	1	− 0.37
SST_Central STB_							0	0.63 ± 0.07	1	− 0.41
SST_hydrophone_									0	− 0.37

Lag values are reported in weeks, and the autocorrelation function (ACF) measures the strength of the correlation (between − 1 and 1) between timeseries at that lag value. Cross-correlations were run for the months of October–March for each year between 2010 and 2018, and ACF values are reported as the mean and standard error for all cross-correlations. D call density was only recorded in one year (October 2016–March 2017), so cross-correlation results are only reported for that year.

### Timing of blue whale aggregations

**Figure 4:**
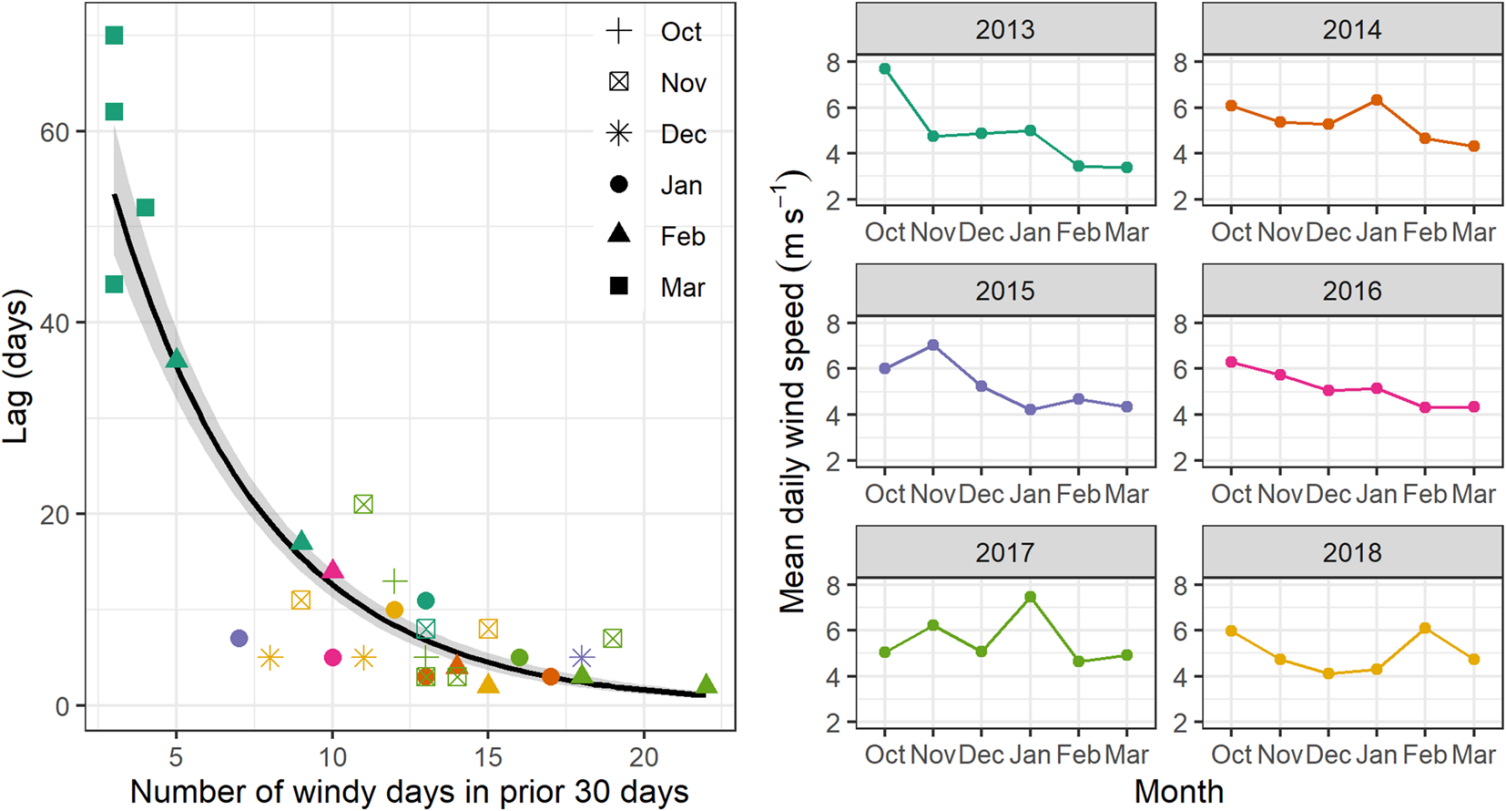
Left panel: the lag between wind events and blue whale aggregations vs. the number of days with wind speed above 5.5 m s^−1^ in the preceding 30 days to the aggregation. Each point represents a blue whale aggregation, symbolized by month and colored by year. The black line represents the fitted relationship using a generalized linear model with a Poisson distribution. Right panels: mean daily wind speed recorded at the Farewell Spit weather station for each month in the spring–summer. The labeled year of each plot represents the year during January.

Wind events, defined as two or more consecutive days with wind speeds > 5.5 m s^−1^, occurred throughout the spring–summer, with a mean of 18 wind events per season between 2009 and 2019. Wind events lasted a mean of three days in duration (range = 2–8 days), with a mean of seven days between events (range = 2–38 days). Wind speed was typically strongest at the start of the season, followed by a decrease in wind speed until another peak in January or February, and then a subsequent decrease over the rest of the summer (Fig. 4). A total of 34 blue whale aggregations were identified from compiled sightings data between 2013 and 2019 (Supplementary Information, Fig. S5). The lag time between wind events and blue whale sighting aggregations was 2.02 ± 0.43 weeks. There was a significant negative relationship between lag time and the number of days with wind speeds > 5.5 m s^−1^ in the preceding 30 days (GLM, χ^2^_1_ = 411.95, p = 2.2*10^–16^, Fig. 4). This result indicates that when there was more wind input in the 30 days prior to a blue whale aggregation formation (*i.e.*, the system was well-mixed), the lag time between the start of a proximate wind event to the start of a blue whale aggregation was shorter. Selection of different wind speed thresholds for the definition of a wind event did influence the GLM result, with increasing wind speed thresholds leading to greater lag times due to a reduced number of identified wind events (Supplementary Information, Fig. S6, Table S2). A comparative assessment of GLM results using different wind speed thresholds confirmed that application of the mean wind speed value of 5.5 m s^−1^ as the threshold to define wind events produces the best fit between lag time and the number of windy days prior to a blue whale aggregation.

## Discussion

Correlative assessments of the relationships between habitat and species distribution patterns are common, yet few studies address the inherent lags between physical drivers and species occurrence^5,28^. Understanding ecosystem function often hinges on quantifying the relationships between physical drivers and biological response, and spatial and temporal lags between these variables should be accounted for, particularly in dynamic marine ecosystems. Without incorporating such lags into ecological understanding, derived management scenarios that rely on predicted species distribution information will lack precision and effectiveness. Our ability to quantify temporal lags between wind, SST, and blue whale occurrence in the STB region of New Zealand will improve the predictive capacity of species distribution models of blue whales to mitigate anthropogenic pressures in this dynamic ecosystem^21,42,43^.

The timeseries cross-correlation results captured lag along the typical pathway of the upwelling plume through the STB region (Fig. 3, Table 1). Decreased SST at Kahurangi shoals, considered to be the source of the upwelling plume, followed increased wind speed with a 1-week lag. SST was correlated between all boxes with a lag time of less than a week, indicating that once the plume forms, the cold-water signal is quickly evident throughout the region. D call density was correlated with decreased SST at the location of the focal hydrophone (MARU2) with a lag of less than a week, but D call density lagged decreased SST at the Central STB and Cape Farewell boxes by one week. Ultimately, D call density lagged increased wind speed at the upwelling source by three weeks. The lag relationships illustrated by this cross-correlation analysis reflect the path of the upwelling plume; locations nearer in space had shorter lag times than more distant spatial locations. Prior research has documented decreased temperature signals indicative of upwelling at the upwelling source at Kahurangi shoals and along the full path of the plume, and elevated primary productivity at more downstream locations^39^. In this study, we quantify these spatial and temporal lags, and expand the biological coupling of these physical oceanographic drivers to blue whale foraging opportunities.

The mean lag time between wind events and blue whale aggregations was 2.02 ± 0.43 weeks across the seven-year time period examined. This estimate is shorter than the results of the timeseries cross-correlation analysis, which revealed that the strongest correlation between wind speed and D call density at the hydrophone location occurred at a 3-week lag. One possible reason for this discrepancy is that the hydrophone was located downstream along the upwelling plume’s path, while the blue whale sighting aggregations were identified throughout the STB region. Indeed, the blue whale aggregations observed during the 2016–2017 season concurrent with the acoustic recordings were beyond the detection range of the hydrophone, and located between the hydrophone location and the upwelling source (Supplementary Information, Fig. S7). Given what is known about the path of the plume and the cross-correlation analysis of lag between wind speed and SST at the different box locations, it is unsurprising that the lag between wind events and whale aggregations would be shorter for locations nearer to the upwelling source, and longer for locations downstream along the plume. Furthermore, D call density was only calculated from acoustic recordings from one year, whereas the timing of aggregations was evaluated over a much longer period.

This study takes a quantitative approach to explicitly test links and quantify lags between the physical driver of upwelling and the downstream biological impacts on a marine predator. Previous work in upwelling systems has examined links between physical forcing and biological responses by primary producers and lower trophic level organisms^32,35,65,66^, or made broad-scale observations about seasonal patterns at higher trophic levels^16,24,30^. For example, in the California current ecosystem, peak krill densities were found to lag the seasonal peak in primary production by three to four months, and blue whales were found downstream of upwelling centers^24^. Furthermore, krill biomass in the California current ecosystem is a primary driver of variability in blue whale migration timing^30^. Our study centered on blue whales in New Zealand extends prior findings by examining the lags along the mechanistic pathway from wind to whales within a season, refining the resolution to isolate individual upwelling events. This result was accomplished first by removing the trend and seasonal components for the timeseries analysis, and then analyzing wind on a per-event basis in relation to blue whale aggregations. Our work demonstrates that blue whale foraging opportunities appear to follow upwelling events by two to three weeks, highlighting the value of examining lag on a scale finer than broad, seasonal cycles. The resolved temporal scale of the lag analyses presented here can improve predictions of blue whale distribution patterns in relation to wind patterns. Fine-scale lag information can enhance model forecasting abilities and improve spatial management approaches to reduce anthropogenic threats to whales such as ship strike risk, fisheries entanglement, or noise impacts.

In addition to quantifying the typical lag times between wind and blue whale aggregations, we also examined the variation in lag times (Fig. 4). Our finding that the amount of prior wind input into the system influences the lag times can further improve and refine predictions of blue whale distribution patterns. With more wind input, the water column is more likely to already be well-mixed, with a preexisting standing stock of available nutrients and enhanced primary productivity. Wind events in an already well-mixed system may aggregate zooplankton on a shorter time scale^66^ and make prey more readily available to blue whales immediately following upwelling, in contrast to the longer time lag needed to spur primary productivity in a more stratified water column. The blue whale aggregations with the longest lag time since the preceding wind event took place in March 2013, when the mean daily wind speed was only 3.39 m s^−1^ and had also been low in the preceding months (Fig. 4). In contrast, some of the shortest lag times between wind events and aggregations took place in January and February 2017, which was the year with the greatest mean daily wind speeds in the summer months (Fig. 4).

While our results are specific to blue whales in the STB, the methodological approach taken in this study is broadly transferrable to other regions, ecosystems, and taxa where a measurable physical driver instigates a pathway of biological response and ultimately impacts predator distribution patterns. Using remotely sensed environmental data, species occurrence information, and timeseries analysis to examine lag between metrics at various spatial locations allows for a quantitative examination of the lags along a mechanistic pathway. Filling data gaps on lags can improve predictive species distribution models, and our findings can potentially be utilized for the conservation of New Zealand blue whales that rely on a dynamic upwelling system in the STB for foraging needs. Furthermore, the knowledge gained from understanding lags can facilitate added lead time for management decisions and ultimately enhance species conservation efforts.

## Supplementary Information

Supplementary Information.

## Data Availability

Satellite SST data were acquired from the ERDDAP server (https://coastwatch.pfeg.noaa.gov/erddap/index.html), wind data were downloaded from New Zealand’s National Climate Database (https://cliflo.niwa.co.nz/), and blue whale sighting information were requested from the New Zealand Department of Conservation marine mammal sightings database (https://www.doc.govt.nz/marine-mammal-sighting-form). All processed data included in this manuscript and data analysis code are available via the Figshare digital repository: https://figshare.com/projects/Temporal_and_spatial_lags_between_wind_coastal_upwelling_and_blue_whale_occurrence/91505.

## References

[1] Mann KH, Lazier JRN (1996). Dynamics of marine ecosystems: Biological-physical interactions in the oceans. Blackwell Sci. Publ..

[2] Ryther J (1969). Photosynthesis and fish production in the sea. Science.

[3] Cushing DH (1990). Plankton production and year-class strength in fish populations: An update of the match/mismatch hypothesis. Adv. Mar. Biol..

[4] Ekman, V. W. On the influence of the earth’s rotation on ocean-currents. (1905).

[5] Grémillet D (2008). Spatial match-mismatch in the Benguela upwelling zone: Should we expect chlorophyll and sea-surface temperature to predict marine predator distributions?. J. Appl. Ecol..

[6] Block BA (2011). Tracking apex marine predator movements in a dynamic ocean. Nature.

[7] Silber GK (2017). Projecting marine mammal distribution in a changing climate. Front. Mar. Sci..

[8] Maxwell SM (2015). Dynamic ocean management: Defining and conceptualizing real-time management of the ocean. Mar. Policy.

[9] Becker EA (2016). Moving towards dynamic ocean management: How well do modeled ocean products predict species distributions?. Remote Sens..

[10] Hazen EL (2018). A dynamic ocean management tool to reduce bycatch and support sustainable fisheries. Sci. Adv..

[11] Abrahms B (2019). Dynamic ensemble models to predict distributions and anthropogenic risk exposure for highly mobile species. Divers. Distrib..

[12] Oestreich WK, Chapman MS, Crowder LB (2020). A comparative analysis of dynamic management in marine and terrestrial systems. Front. Ecol. Environ..

[13] Redfern JV (2006). Techniques for cetacean-habitat modeling. Mar. Ecol. Prog. Ser..

[14] Becker EA (2010). Comparing California current cetacean-habitat models developed using in situ and remotely sensed sea surface temperature data. Mar. Ecol. Prog. Ser..

[15] Palacios DM (2019). Ecological correlates of blue whale movement behavior and its predictability in the California Current Ecosystem during the summer-fall feeding season. Mov. Ecol..

[16] Thompson SA (2012). Linking predators to seasonality of upwelling: Using food web indicators and path analysis to infer trophic connections. Prog. Oceanogr..

[17] Fiedler PC (1998). Blue whale habitat and prey in the California Channel Islands. Deep Res. Part II Top. Stud. Oceanogr..

[18] Buchan SJ, Quiñones RA (2016). First insights into the oceanographic characteristics of a blue whale feeding ground in northern Patagonia, Chile. Mar. Ecol. Prog. Ser..

[19] Gill PC (2011). Blue whale habitat selection and within-season distribution in a regional upwelling system off southern Australia. Mar. Ecol. Prog. Ser..

[20] de Vos A, Pattiaratchi CB, Wijeratne EMS (2013). Surface circulation and upwelling patterns around Sri Lanka. Biogeosciences Discuss..

[21] Barlow DR, Bernard KS, Escobar-Flores P, Palacios DM, Torres LG (2020). Links in the trophic chain: Modeling functional relationships between in situ oceanography, krill, and blue whale distribution under different oceanographic regimes. Mar. Ecol. Prog. Ser..

[22] Williams TM, Haun J, Davis RW, Fuiman LA, Kohin S (2001). A killer appetite: Metabolic consequences of carnivory in marine mammals. Comp. Biochem. Physiol. Part A.

[23] Goldbogen JA (2011). Mechanics, hydrodynamics and energetics of blue whale lunge feeding: Efficiency dependence on krill density. J. Exp. Biol..

[24] Croll DA (2005). From wind to whales: Trophic links in a coastal upwelling system. Mar. Ecol. Prog. Ser..

[25] Hazen EL, Friedlaender AS, Goldbogen JA (2015). Blue whales (Balaenoptera musculus) optimize foraging efficiency by balancing oxygen use and energy gain as a function of prey density. Sci. Adv..

[26] Nickels CF, Sala LM, Ohman MD (2018). The morphology of euphausiid mandibles used to assess selective predation by blue whales in the southern sector of the California Current System. J. Crustac. Biol..

[27] Abrahms B (2019). Memory and resource tracking drive blue whale migrations. Proc. Natl. Acad. Sci. USA.

[28] Visser F, Hartman KL, Pierce GJ, Valavanis VD, Huisman J (2011). Timing of migratory baleen whales at the azores in relation to the north atlantic spring bloom. Mar. Ecol. Prog. Ser..

[29] Fossette S (2017). Resource partitioning facilitates coexistence in sympatric cetaceans in the California Current. Ecol. Evol..

[30] Szesciorka AR (2020). Timing is everything: Drivers of interannual variability in blue whale migration. Sci. Rep..

[31] Botsford LW, Lawrence CA, Dever EP, Hastings A, Largier J (2003). Wind strength and biological productivity in upwelling systems: An idealized study. Fish. Oceanogr..

[32] Botsford LW, Lawrence CA, Dever EP, Hastings A, Largier J (2006). Effects of variable winds on biological productivity on continental shelves in coastal upwelling systems. Deep Res. Part II Top. Stud. Oceanogr..

[33] Cury P, Roy C (1989). Optimal environmental window and pelagic fish recruitment success in upwelling areas. Can. J. Fish. Aquat. Sci..

[34] Yokomizo H, Botsford LW, Holland MD, Lawrence CA, Hastings A (2010). Optimal wind patterns for biological production in shelf ecosystems driven by coastal upwelling. Theor. Ecol..

[35] Nieblas AE, Sloyan BM, Hobday AJ, Coleman R, Richardson AJ (2009). Variability of biological production in low wind-forced regional upwelling systems: A case study off southeastern Australia. Limnol. Oceanogr..

[36] Stevens CL, O’Callaghan JM, Chiswell SM, Hadfield MG (2019). Physical oceanography of New Zealand/Aotearoa shelf seas: A review. N. Z. J. Mar. Freshw. Res..

[37] Heath RR, Gilmour AE (1987). Flow and hydrological variability in the Kahurangi plume off north-west South Island, New Zealand. N. Z. J. Mar. Freshw. Res..

[38] Shirtcliffe TGL (1990). Dynamics of the Cape Farewell upwelling plume, New Zealand. N. Z. J. Mar. Freshw. Res..

[39] Chiswell SM, Zeldis JR, Hadfield MG, Pinkerton MH (2017). Wind-driven upwelling and surface chlorophyll blooms in Greater Cook Strait. N. Z. J. Mar. Freshw. Res..

[40] Bradford-Grieve JM, Murdoch RC, Chapman BE (1993). Composition of macrozooplankton assemblages associated with the formation and decay of pulses within an upwelling plume in greater cook strait, New Zealand. N. Z. J. Mar. Freshw. Res..

[41] Bradford JM, Chapman B (1988). Nyctiphanes australis (euphausiacea) and an upwelling plume in Western Cook Strait, New Zealand. N. Z. J. Mar. Freshw. Res..

[42] Torres LG (2013). Evidence for an unrecognised blue whale foraging ground in New Zealand. N. Z. J. Mar. Freshw. Res..

[43] Barlow DR (2018). Documentation of a New Zealand blue whale population based on multiple lines of evidence. Endanger. Species Res..

[44] Torres LG, Barlow DR, Chandler TE, Burnett JD (2020). Insight into the kinematics of blue whale surface foraging through drone observations and prey data. PeerJ.

[45] Seers, B. & Shears, N. *New Zealand’s Climate Data in R: An Introduction to clifro*. (2015).

[46] Tsagris, M., Athineou, G., Sajib, A., Amson, E. & Waldstein, M. Directional: Directional Statistics. R package version 4.3. (2020).

[47] Mendelssohn, R. *rerddapXtracto: Extracts Environmental Data from ‘ERDDAP’ Web Services*. (2019).

[48] Calupca TA, Fristrup KM, Clark CW (2000). A compact digital recording system for autonomous bioacoustic monitoring. J. Acoust. Soc. Am..

[49] Oleson EM (2007). Behavioral context of call production by eastern North Pacific blue whales. Mar. Ecol. Prog. Ser..

[50] McDonald MA (2006). An acoustic survey of baleen whales off Great Barrier Island, New Zealand. N. Z. J. Mar. Freshw. Res..

[51] Mellinger DK, Clark CW (2000). Recognizing transient low-frequency whale sounds by spectrogram correlation. J. Acoust. Soc. Am..

[52] Center for Conservation Bioacoustics. Raven Pro: Interactive Sound Analysis Software. (2019).

[53] Mellinger DK, Roch MA, Nosal E-M, Klinck H, Au WWL, Lammers MO (2016). Signal processing. Listening in the Ocean.

[54] Fregosi S (2020). Comparison of fin whale 20 Hz call detections by deep-water mobile autonomous and stationary recorders. J. Acoust. Soc. Am..

[55] Collins MD (1993). A split-step Pade solution for the parabolic equation method. J. Acoust. Soc. Am..

[56] Amante, C. & Eakins, B. W. *ETOPO1 1 Arc-Minute Global Relief Model: Procedures, Data Sources and Analysis*. *NOAA Technical Memorandum NESDIS NGDC-24* (2009).

[57] Wentworth CK (1922). A scale of grade and class terms for clastic sediments. J. Geol..

[58] Bostock H (2019). Distribution of surficial sediments in the ocean around New Zealand/Aotearoa. Part B: continental shelf. N. Z. J. Geol. Geophys..

[59] Samaran F, Guinet C, Adam O, Motsch J-F, Cansi Y (2010). Source level estimation of two blue whale subspecies in southwestern Indian Ocean. J. Acoust. Soc. Am..

[60] Holmes EE, Scheuerell MD, Ward EJ (2018). Applied Time Series Analysis for Fisheries and Environmental Data.

[61] Chiswell SM, Sutton PJH (2020). Relationships between long-term ocean warming, marine heat waves and primary production in the New Zealand region. N. Z. J. Mar. Freshw. Res..

[62] Santora JA, Reiss CS, Loeb VJ, Veit RR (2010). Spatial association between hotspots of baleen whales and demographic patterns of Antarctic krill Euphausia superba suggests size-dependent predation. Mar. Ecol. Prog. Ser..

[63] Goetz, K. T. *et al. Satellite Tracking of Blue Whales in New Zealand Waters, 2018 Voyage Report*. (2018).

[64] Double, M. C. *et al.* Cruise report of the 2013 Antarctic blue whale voyage of the Southern Ocean Research Partnership. *Int. Whal. Comm. SC/65a/SH21* 1–16 (2013).

[65] Chenillat F, Rivière P, Capet X, Franks PJS, Blanke B (2013). California coastal upwelling onset variability: Cross-shore and bottom-up propagation in the planktonic ecosystem. PLoS ONE.

[66] Benoit-Bird KJ, Waluk CM, Ryan JP (2019). Forage species swarm in response to coastal upwelling. Geophys. Res. Lett..

