# Supplementary material for: Temporal and spatial lags between wind, coastal upwelling, and blue whale occurrence
Source: Sci Rep. 2021 Mar 25;11:6915. doi: 10.1038/s41598-021-86403-y (PMC7994810; doi:10.1038/s41598-021-86403-y)
Supplement: Supplementary file 1 — Supplementary Information. [file 41598_2021_86403_MOESM1_ESM.pdf]

Temporal and spatial lags between wind, coastal upwelling, and blue whale occurrence

Dawn R. Barlow[1]*, Holger Klinck[2,3], Dimitri Ponirakis[2], Christina Garvey[4], Leigh G. Torres[1]

[1]Geospatial Ecology of Marine Megafauna Lab, Marine Mammal Institute, and Department of Fisheries and Wildlife, Oregon State University, Newport, Oregon, USA
[2]Center for Conservation Bioacoustics, Cornell University, Ithaca, New York, USA
[3]Marine Mammal Institute, Department of Fisheries and Wildlife, Oregon State University, Newport, Oregon, USA
[4]University of Maryland, College Park, Maryland, USA

*dawn.barlow@oregonstate.edu

Supplementary Information

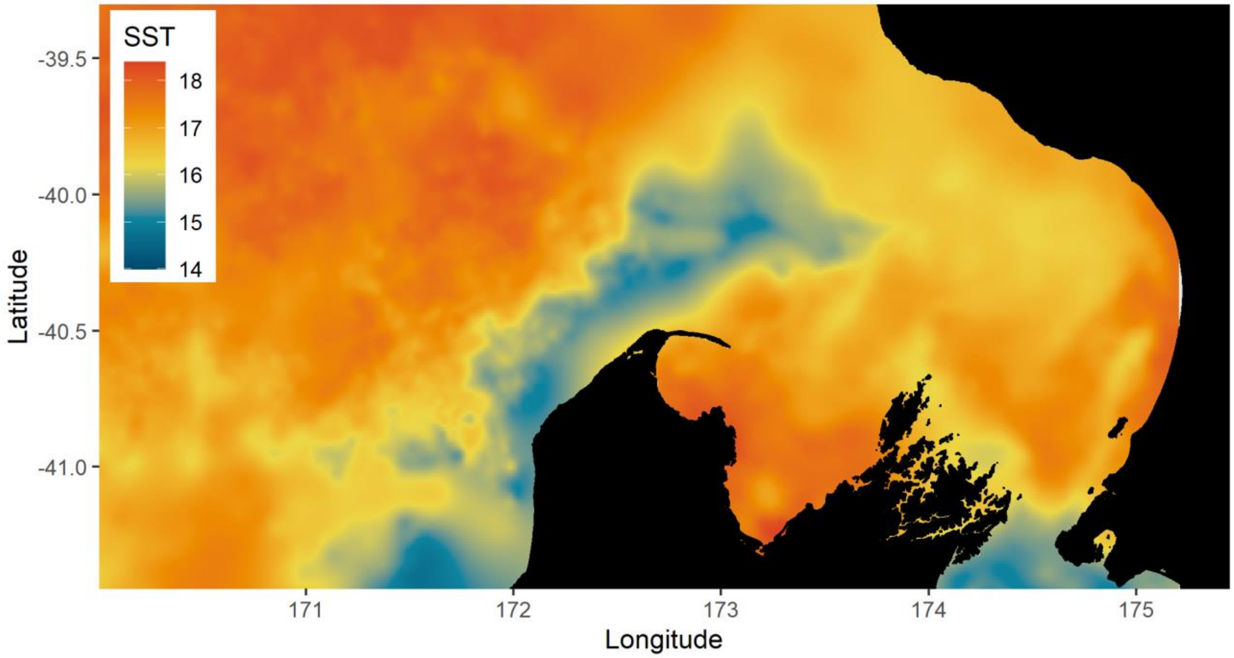

Figure S1. Example daily Multi-scale Ultra-high Resolution (MUR; https://podaac.jpl.nasa.gov/dataset/MUR-JPL-L4-GLOB-v4.1) satellite image of sea surface temperature (SST) from 24 January 2014, illustrating the surface signature of the cold water upwelling plume originating off Kahurangi shoals and extending north and eastward into the South Taranaki Bight region.

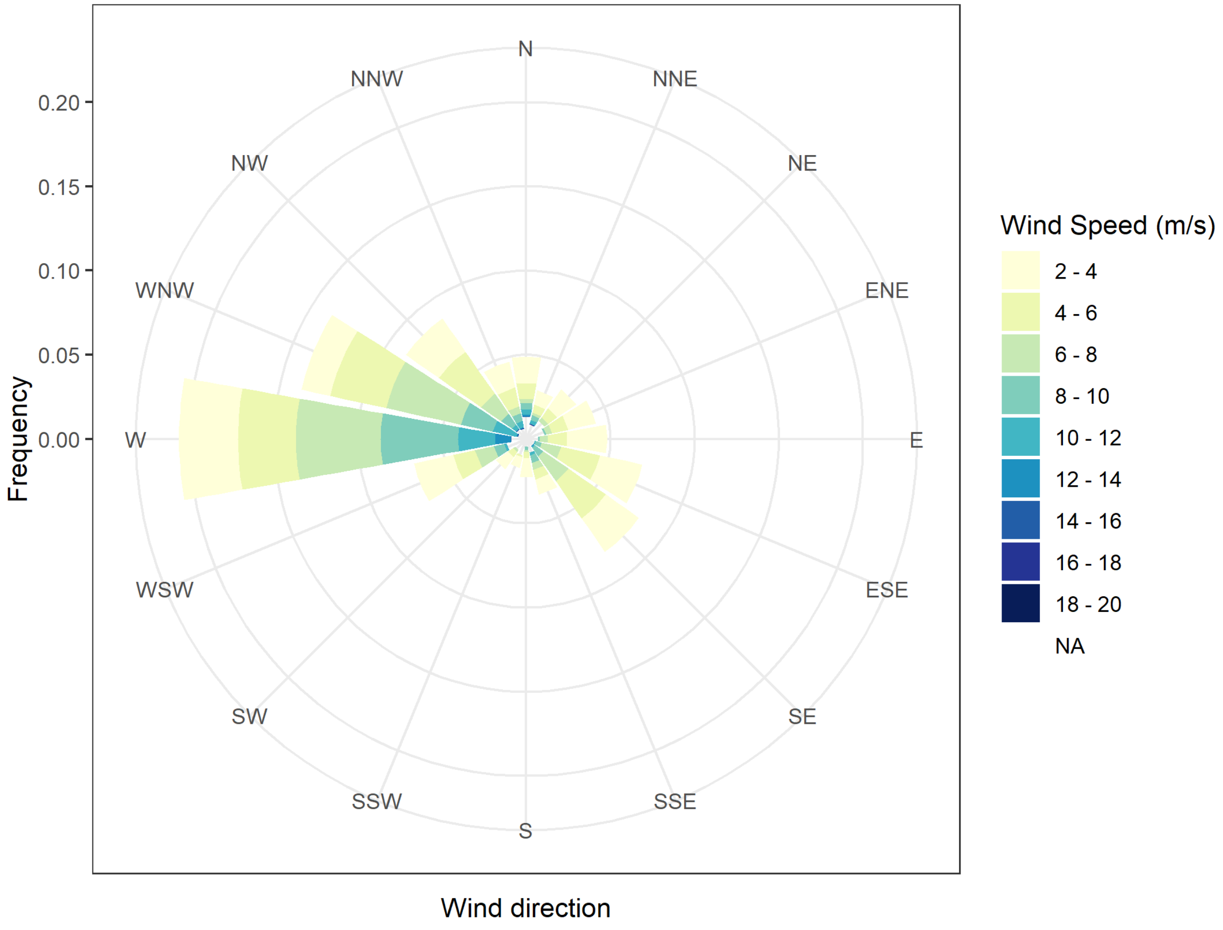

Figure S2. Rose plot showing the frequency of wind speed and direction recorded at the Farewell Spit weather station during the months of October-March between 2009 and 2019.

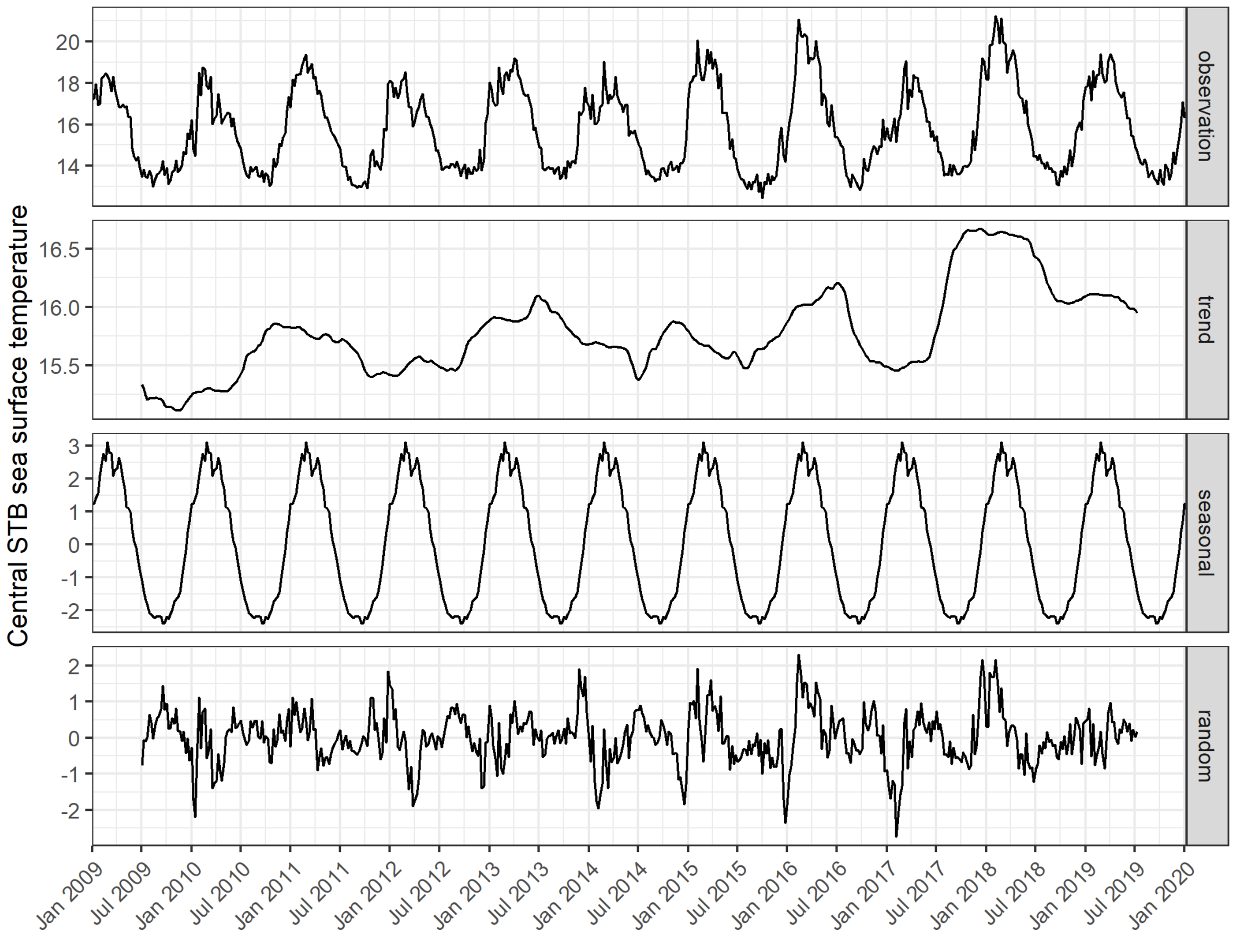

Figure S3. Example decomposition of an additive timeseries. Sea surface temperature at the Central STB sampling location between 2009 and 2019. The observed time series (values measured in ºC) is on the top panel, and the trend, seasonal, and random components of the timeseries are shown below.

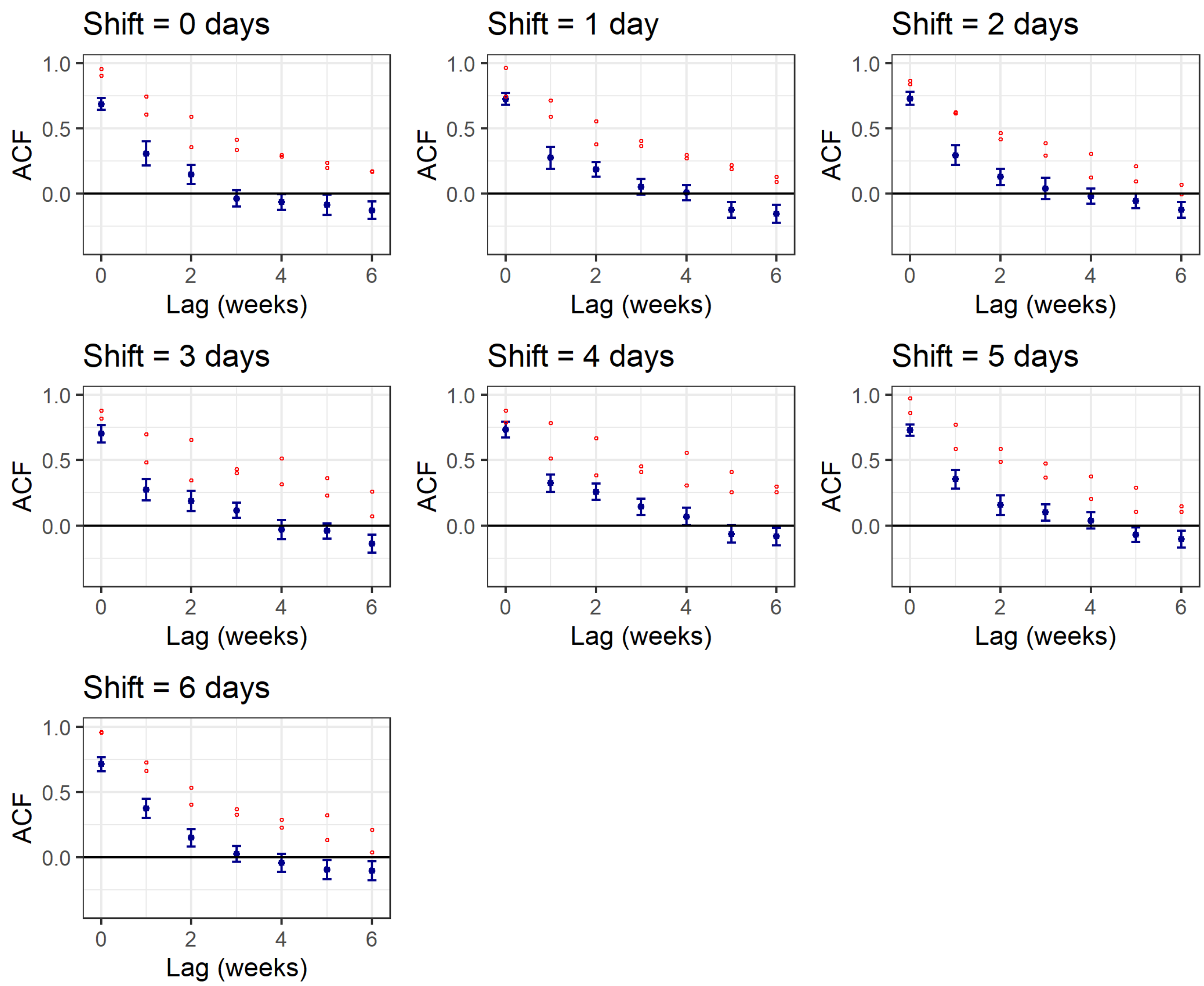

Figure S4. Results from the timeseries cross-correlation analysis between SST at Kahurangi Shoals and SST at Cape Farewell (see Fig. 1 in the main text for geographic location) during the austral spring-summer season. The cross-correlations were conducted on a weekly timeseries, whereby one daily SST measurement was selected per week (*i.e.* measurement taken every 7th day). To investigate whether the start day for weekly subsampling might impact the results, start date was shifted by one day for each of the 7 days of the week and the outcome was compared. The resulting plots illustrate that, while there is minute observable change by shifting the subsampling start date, the impact on the outcome of the cross-correlation is negligible (highest ACF value remains at lag = 0 weeks in all cases). Therefore, weekly subsampling where shift = 0 is applied for the remainder of the analyses in this study. Lag values are reported in weeks (x-axes). The autocorrelation function (ACF) measures the strength of the correlation between time series at that lag value, with values > 0 representing positive correlation, and values < 0 representing negative correlation (y-axes). Mean ACF was calculated at each lag step for all years between 2010 and 2018, with 2016 and 2018 partitioned *a priori* because of documented marine heatwave conditions. The ACF values are plotted in blue for "typical" conditions (mean ± standard error), and values from known heatwave years are plotted in red.

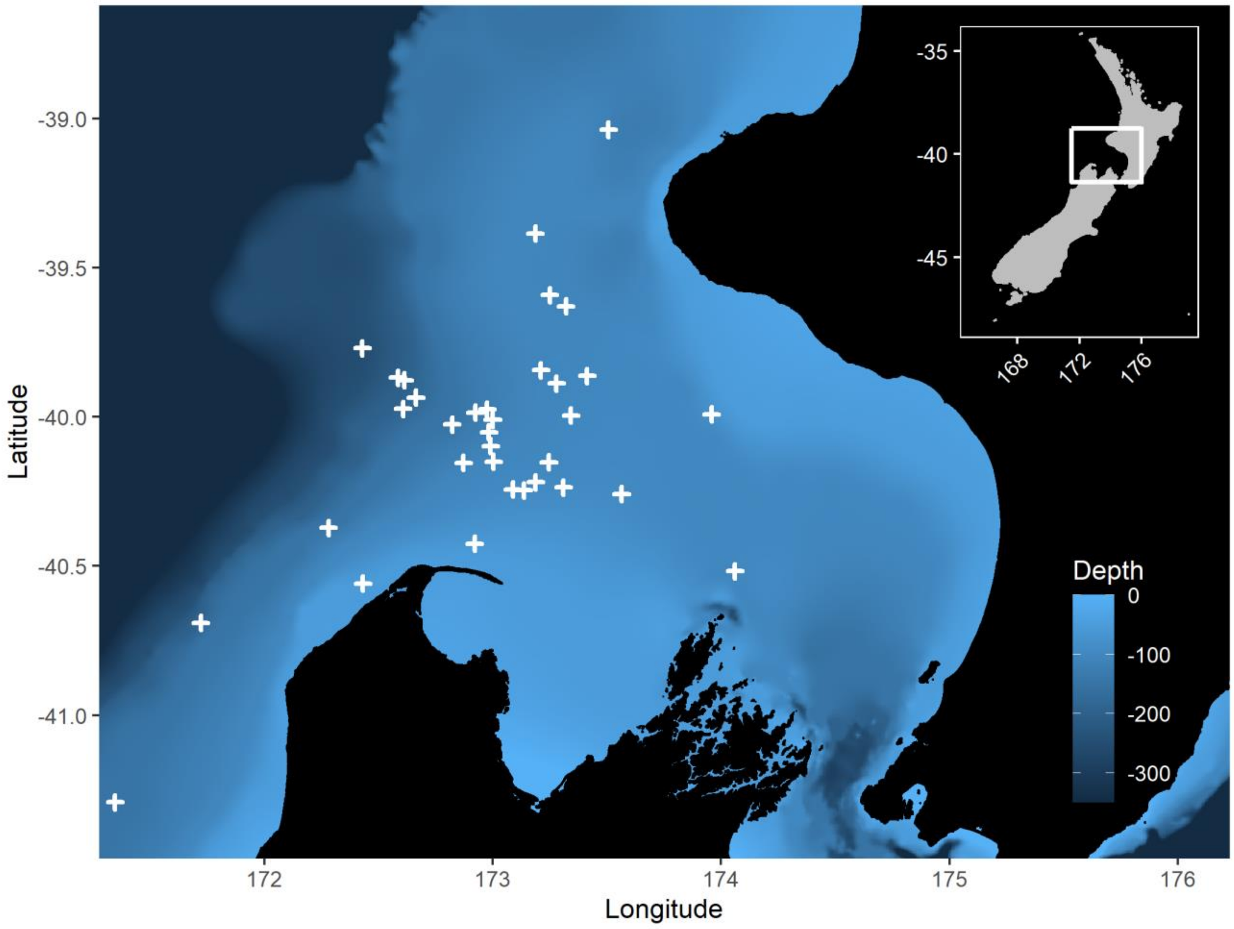

Figure S5. Spatial locations of all blue whale sighting aggregations (≥ 5 whales reported within one week, within 50 km) identified within the South Taranaki Bight region between 2013 and 2019 from sighting reports. Aggregation centroid locations are denoted by the white crosses. Location of the study area within New Zealand is denoted by the white rectangle in the upper right panel.

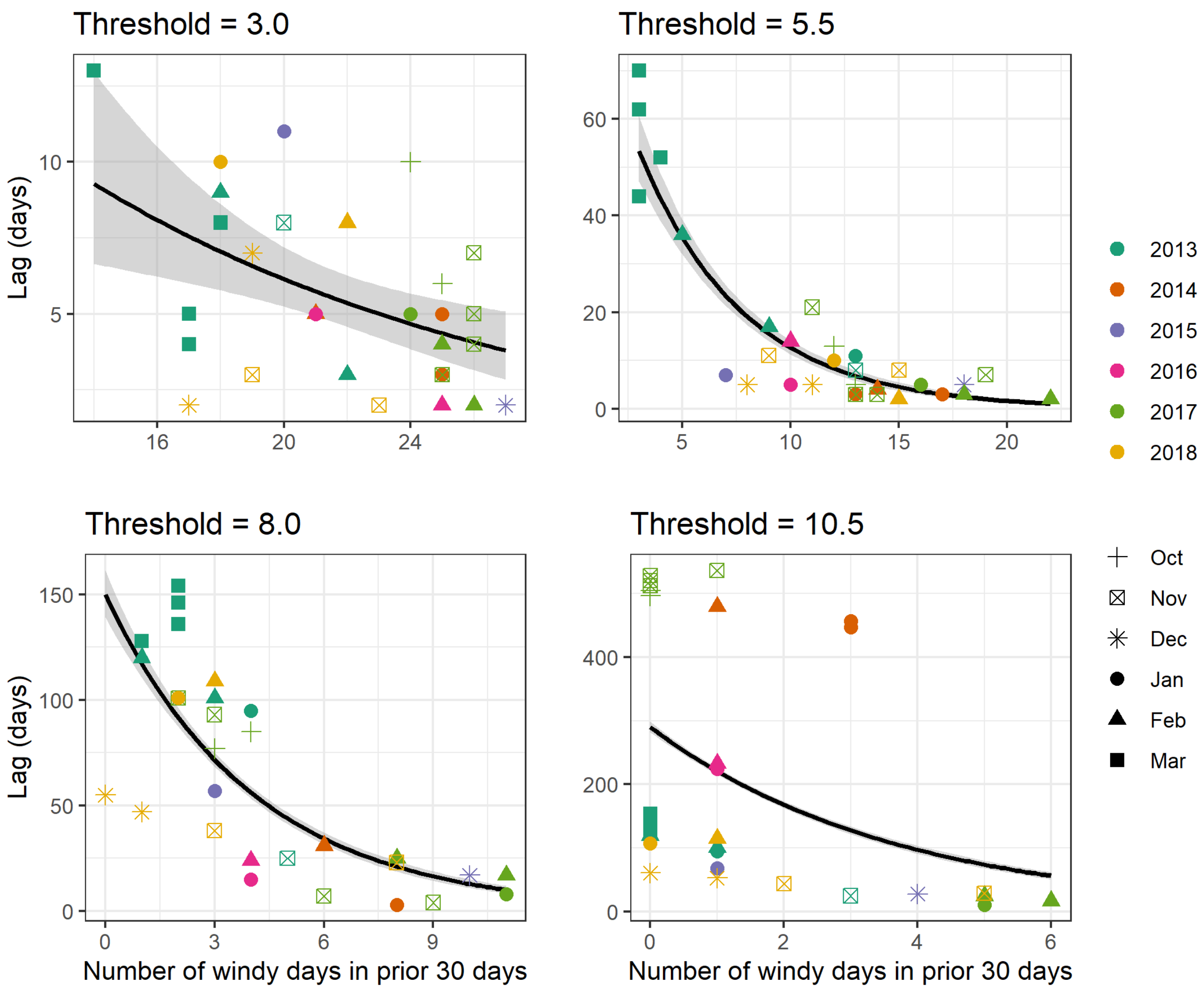

Figure S6. Plots comparing the lag between wind events and blue whale aggregations *vs.* the number of days where wind speed exceeded the selected threshold in the preceding 30 days to the aggregation. Threshold values are the wind speed measured in m s^{-1}, used to define wind events. Each point represents a blue whale aggregation, symbolized by month and year. The lines represent the fitted relationship using a generalized linear model (GLM) with a Poisson distribution. The GLM fit was determined to be optimal using a threshold of 5.5 m s^{-1} (Table S2), and thus applied in the study. Note the different x- and y-axis ranges in each panel.

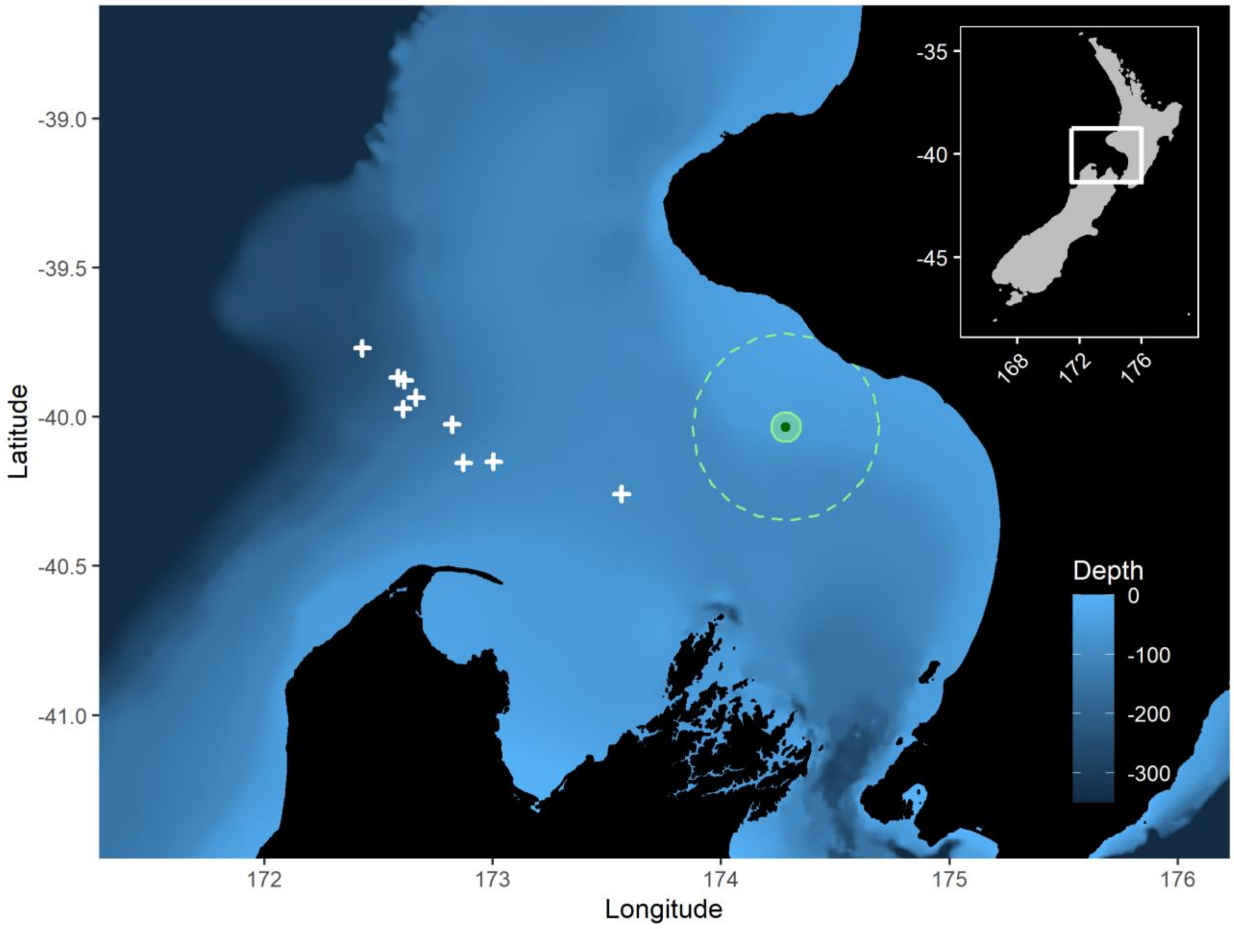

Figure S7. Blue whale aggregations (white crosses) in the spring-summer 2016-2017 season that were observed during the concurrent hydrophone recording period. The hydrophone location is indicated by the dark green point. Mean daily detection range (5.5 km) around the hydrophone is shown by the light green circle, and the maximum daily detection range (34.8 km) is indicated by the light green dashed line. The lag time between wind events and these blue whale aggregations during the hydrophone recording period ranged between 2-21 days, with a mean of 7 days.

Table S1. Results from the cross-correlation analysis between timeseries data from all assess location boxes. Lag values are reported in weeks, and the autocorrelation function (ACF) measures the strength of the correlation between time series at that lag value. Cross-correlations were run for the months of October-March for each year between 2010 and 2018, and ACF values are reported as the mean and standard error for all cross-correlations. D call density was only recorded in one year (October 2016-March 2017), so cross-correlation results are only reported for that year.

	SST$_{Kahurangi}$		SST$_{Cape\ Farewell}$		SST$_{Central\ STB}$		SST$_{MARU5}$		SST$_{MARU4}$		SST$_{MARU2}$ (focal hydrophone)		SST$_{MARU3}$		SST$_{MARU1}$		Dcalls $_{MARU2}$ (focal hydrophone)	
	**Lag**	**ACF**	**Lag**	**ACF**	**Lag**	**ACF**	**Lag**	**ACF**	**Lag**	**ACF**	**Lag**	**ACF**	**Lag**	**ACF**	**Lag**	**ACF**	**Lag**	**ACF**
**Wind speed**	1	-0.17 ± 0.04	2	-0.23 ± 0.05	2	-0.25 ± 0.06	1	-0.23 ± 0.08	1	-0.22 ± 0.05	2	-0.21 ± 0.07	1	-0.16 ± 0.05	1	-0.24 ± 0.07	3	0.19
**SST$_{Kahurangi}$**			0	0.69 ± 0.04	0	0.57 ± 0.05			0	0.46 ± 0.10	0	0.49 ± 0.08	0	0.50 ± 0.08	0	0.44 ± 0.08	3	-0.32
**SST$_{Cape\ Farewell}$**					0	0.60 ± 0.07			0	0.57 ± 0.09	0	0.53 ± 0.07	0	0.49 ± 0.09	0	0.45 ± 0.08	1	-0.37
**SST$_{Central\ STB}$**									0	0.54 ± 0.07	0	0.63 ± 0.07	0	0.51 ± 0.07	0	0.53 ± 0.10	1	-0.41
**SST$_{MARU5}$**									0	0.48 ± 0.06	0	0.56 ± 0.07	0	0.45 ± 0.07	0	0.49 ± 0.07	3	-0.42
**SST$_{MARU4}$**											0	0.55 ± 0.04	0	0.51 ± 0.04			0	-0.51
**SST$_{MARU2}$ (focal hydrophone)**									0	0.55 ± 0.04			0	0.62 ± 0.07			0	-0.37
**SST$_{MARU3}$**																		
**SST$_{MARU1}$**																		

Table S2. Results of a sensitivity analysis comparing the impact of selecting different wind speed thresholds for defining wind events. Lag measures the time between blue whale aggregations and the previous wind event. A generalized linear model (GLM) with a Poisson distribution was fit between lag and the number of days with wind speeds above the selected wind threshold in the preceding 30 days to the aggregation. Models were compared using the deviance between observed and expected values (χ^2), significance of the fitted relationship (p), and model performance (measured using Akaike's Information Criterion, AIC[1]) to evaluate the impact of selecting different wind thresholds. Taking into consideration the increasing AIC at higher wind thresholds and the lower p-value at the 3.0 m s^{-1} threshold, the wind speed threshold of 5.5 m s^{-1} was maintained for analysis.

**Wind threshold (m s^{-1})**	**Mean lag ± standard error (weeks)**	**GLM results**		
		**χ^2 (deviance)**	**p**	**AIC**
3.0	0.84 ± 0.07	10.162	0.001	142.23
5.5	2.02 ± 0.43	411.95	$2.2*10^{-16}$	211.12
8.0	8.19 ± 1.16	730.47	$2.2*10^{-16}$	631.10
10.5	31.89 ± 4.82	1039.00	$2.2*10^{-16}$	4444.9

References:

1. Akaike, H. Akaike's Information Criterion. in *International Encyclopedia of Statistical Science* (2011). doi:10.1007/978-3-642-04898-2_110
